# Noncoding RNAs in rheumatoid arthritis: modulators of the NF-κB signaling pathway and therapeutic implications

**DOI:** 10.3389/fimmu.2024.1486476

**Published:** 2024-10-28

**Authors:** Dina Seyedi, Najmadin Espandar, Maryam Hojatizadeh, Yaser Mohammadi, Farzad Sadri, Zohreh Rezaei

**Affiliations:** ^1^ School of Medicine, Tehran University of Medical Sciences, Tehran, Iran; ^2^ Department of Exercise Physiology and Corrective Exercises, Faculty of Sport Sciences, Urmia University, Urmia, Iran; ^3^ Department of Immunology, School of Public Health, Tehran University of Medical Sciences, Tehran, Iran; ^4^ Department of Biochemistry, School of Medicine, Iran University of Medical Sciences, Tehran, Iran; ^5^ Student Research Committee, Birjand University of Medical Sciences, Birjand, Iran; ^6^ Cellular and Molecular Research Center, Birjand University of Medical Sciences, Birjand, Iran; ^7^ Department of Biology, University of Sistan and Baluchestan, Zahedan, Iran

**Keywords:** noncoding RNAs, NF-κB signaling pathway, rheumatoid arthritis, therapeutic targets, synovial fibroblasts

## Abstract

Rheumatoid arthritis (RA) is a chronic autoimmune disease that causes joint inflammation and gradual tissue destruction. New research has shown how important noncoding RNAs (ncRNAs) are for changing immune and inflammatory pathways, such as the WNT signaling pathway, which is important for activating synovial fibroblasts and osteoblasts to work. This article examines the current understanding of several ncRNAs, such as miRNAs, lncRNAs, and circRNAs, that influence NF-κB signaling in the pathogenesis of RA. We investigate how these ncRNAs impact NF-κB signaling components, altering cell proliferation, differentiation, and death in joint tissues. The paper also looks at how ncRNAs can be used as potential early detection markers and therapeutic targets in RA because they can change important pathogenic pathways. This study highlights the therapeutic potential of targeting ncRNAs in RA therapy techniques, with the goal of reducing inflammation and stopping disease progression. This thorough analysis opens up new possibilities for understanding the molecular foundations of RA and designing novel ncRNA-based treatments.

## Background

Rheumatoid arthritis (RA) is a chronic autoimmune disease characterized by inflammation that progresses. Permanent synovitis triggers the disease, resulting in irreversible joint damage. Individuals with RA frequently suffer bilateral joint soreness, edema, and stiffness. Damage to extra-articular organs and systems may occur as RA worsens. The risk of incidence of cardiovascular illness, a significant and potentially deadly comorbidity, closely correlates with RA disease activity (1). A worldwide health concern, RA affects around 240 out of every 100,000 people. Moreover, the prevalence of RA is rising globally in line with the fast-growing aging population (2). The pathophysiology and etiology of RA are extremely complex and unknown. They involve inheritance, triggers from the environment, and epigenetic alterations (3). The four main characteristics of RA pathology are usually acknowledged to be synovial hyperplasia, ongoing inflammation, articular cartilage deterioration, and bone erosion (4). Recent developments in RA pathophysiology have produced novel biologic and small-molecule inhibitors over the last 20 years. However, due to inadequate therapy, many RA patients continue to suffer from severe disability and a lower quality of life. Thus, to enhance therapeutic results, more understanding of the cellular and molecular pathways behind tissue damage, persistent inflammation, and synovial hyperplasia is required (5).

Non-coding RNAs (ncRNAs) are essential for controlling gene expression and biological processes, as demonstrated by recent developments in molecular biology (6). While not encoding proteins like coding RNAs, non-coding RNAs (ncRNAs) are nonetheless active in several aspects of gene expression during and after transcription (7). NcRNAs are classified into three groups, each having its own mechanism, including microRNAs (miRNAs), long non-coding RNAs (lncRNAs), and circular RNAs (circRNAs) (8, 9).

MicroRNAs are short RNA molecules, measuring approximately 22 nucleotides in length. When miRNAs bind to the 3’ untranslated regions (UTRs) of target mRNAs, they stop translation or destroy the mRNA (Fabian, Sonenberg et al., 2010). Phenotypic scaffolds, guides, or decoys, long non-coding RNAs (>200 nucleotides), influence the dynamics of chromatin and the expression of genes (10). Circular RNAs function as miRNA sponges, trapping miRNAs and avoiding them from attaching to target mRNAs due to their covalently closed loop topologies (11).

Extensive research has demonstrated how important dysregulated NF-κB activation is for the emergence of several autoimmune disorders, such as RA (12, 13). The NF-κB family of inducible transcription factors regulates several genes involved in immunological and inflammatory responses (14). Abnormalities caused by aberrant NF-κB activation in RA lead to long-term inflammation and joint destruction (14).

The aim of this study is to recognize and characterize ncRNAs associated with the NF-κB signaling pathway in RA. Finding out how different ncRNAs, like miRNAs, lncRNAs, and circRNAs, control this pathway is important because NF-κB is a key player in the progress of RA. By investigating these ncRNAs, we hope to gain insights into their specific contributions and interactions within the NF-κB signaling network. This study aims to reveal novel molecular targets for developing more effective RA therapies.

## The complex pathogenesis of rheumatoid arthritis

**Figure 1:**
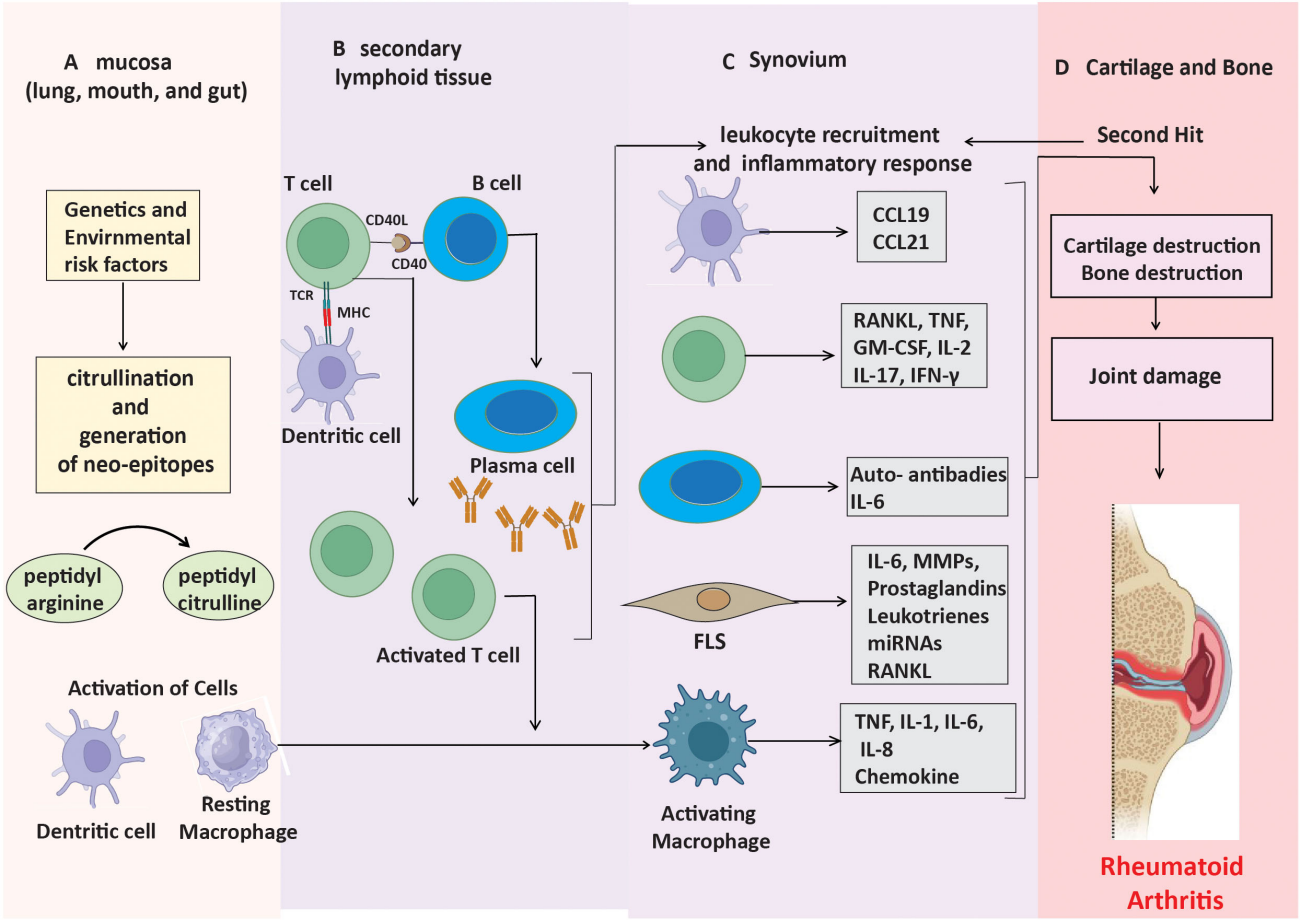
Mechanisms leading to ACPA-positive RA progression. **(A)** Neo-epitopes form in mucosa through post-translational modifications. **(B)** APCs present these peptides, triggering autoantibody production. **(C)** Stromal cells, APCs, and macrophages produce inflammatory factors, driving synovial inflammation. **(D)** Cytokines and immune responses contribute to cartilage and bone destruction.

The presence or absence of anti-citrullinated protein antibodies (ACPAs) divides RA into two main subgroups: ACPA-positive RA and ACPA-negative RA (15). Generally, ACPA-positive rheumatoid arthritis is associated with a more severe disease trajectory, elevated rates of joint erosion, and greater disability compared to ACPA-negative rheumatoid arthritis (16). The two subtypes also differ in terms of their fundamental genetic risk factors and environmental impacts. Environmental variables, including smoking, predispose a genotype to ACPA-positive rheumatoid arthritis (17). Peptidyl arginine deiminases (PADs) mediate a process known as citrullination, which facilitates the transformation of the amino acid arginine into citrulline (18). Certain bacterial pathogens, such as Porphyromonas gingivalis (associated with periodontal disease) and Aggregatibacter actinomycetemcomitans, can also induce this alteration and contribute to the exacerbation of rheumatoid arthritis progression (19). Citrullinated proteins transform into neoantigens upon formation and present themselves to CD4+ T lymphocytes through major histocompatibility complex (MHC) class II molecules, particularly HLA-DRB1 alleles, which significantly correlate with rheumatoid arthritis susceptibility (**Figure 1A**) (20, 21). When T cells are activated and present these modified peptides, they trigger B cells to produce autoantibodies such as ACPAs and RF (**Figure 1B**) (22). These autoantibodies can be identified years before the onset of clinical symptoms, signifying an initial phase of immunological activation.

In the early stages of ACPA-positive rheumatoid arthritis, a lot of CD4+ T cells and macrophages enter the synovium (23). This causes the production of pro-inflammatory cytokines like TNF-α, IL-1, and IL-6. This leads to the activation of synovial fibroblast-like synoviocytes (FLS) and an increased production of matrix metalloproteinases (MMPs), which decompose the extracellular matrix (**Figure 1C**) (24). The paracrine and autocrine functions of cytokines, along with ongoing adaptive immune responses, contribute to disease progression by promoting FLS to adopt an invasive phenotype, which facilitates cartilage degradation and bone erosion (**Figure 1D**). These altered FLS can also move between joints, sustaining inflammation in several joint regions (25).

Even though ACPA-negative rheumatoid arthritis is similar to other types in terms of joint involvement and inflammation, it has its own immune system and molecular features. In contrast to ACPA-positive RA, ACPA-negative RA does not depend on citrullination or the existence of anti-citrullinated antibodies for its pathogenesis. The pathogenesis is instead governed by stromal cells, particularly fibroblast-like synoviocytes, which are crucial in mediating inflammation and advancing disease progression. In healthy synovial tissue, FLS play a crucial role in sustaining the synovial lining and synthesizing proteins essential for joint lubrication (26). On the other hand, in RA, they transform into aggressive pathogenic cells. They learn to express Toll-like receptors (TLRs) and work as antigen-presenting cells (APCs) (27, 28). These stromal cells express antigens via histocompatibility complex (MHC) II receptors and release cytokines and chemokines. Thus, these stromal cells are part of the innate immune system (29). In ACPA-negative RA, cytokines activate endothelial cells, resulting in the recruitment of more immune cells, including Th17 cells and macrophages, into the synovial compartment, thereby intensifying the inflammatory response. Furthermore, infiltrating cells, including fibroblasts, macrophages, T cells, B cells, and plasma cells, stimulated by blood-activated fibroblasts and macrophages, contribute to the pathophysiology of ACPA-negative rheumatoid arthritis (30). These cells create a proinflammatory milieu within the synovium, wherein stromal cells, especially FLS, are crucial in maintaining inflammation and facilitating disease advancement. Activated cells release proinflammatory cytokines, including IL-1, IL-6, IL-17, TNF-α, and MMPs, resulting in the eventual degradation of cartilage and bone (31, 32). Furthermore, activated B cells, with the aid of antigen-presenting cells and Th cells, ultimately undergo differentiation into plasma cells for the purpose of synthesizing and producing diverse immunoglobulins. There is evidence that the release of Th1 cytokines in RA leads to more Th17 cells entering the synovial tissues and the production of IL-17 (33, 34).

**Figure 2:**
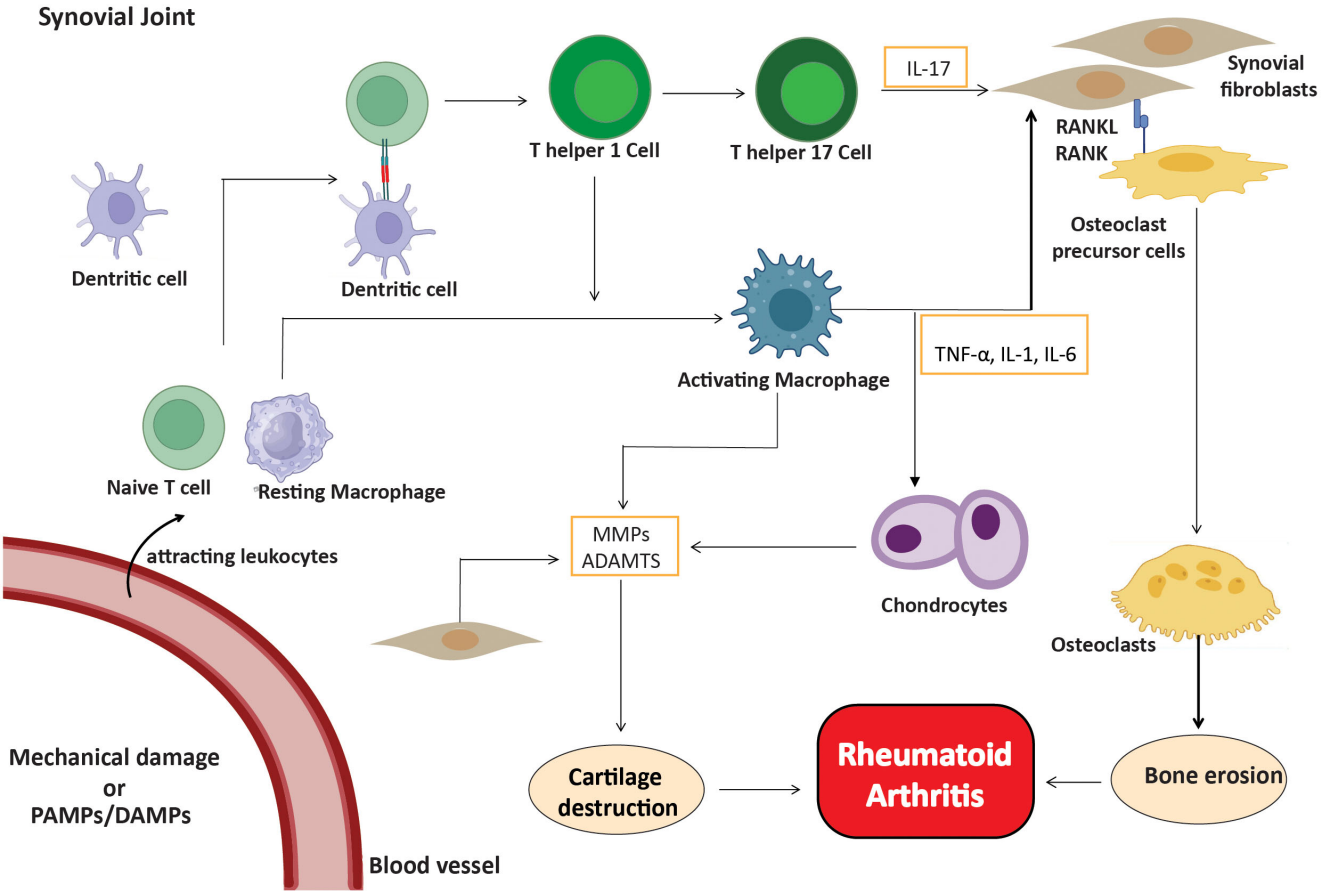
Inflammatory cells in ACPA-negative RA. Fibroblasts, macrophages, T cells, B cells, and plasma cells create a proinflammatory environment, with FLS being crucial. Activated cells release cytokines (IL-1, IL-6, IL-17, TNF-α) and MMPs, leading to cartilage and bone degradation.

As the disease progresses, activated fibroblast-like synoviocytes form direct interactions with dendritic cells, macrophages, and pre-osteoclasts. RANK-RANKL signaling facilitates interactions that enhance osteoclast development and bone resorption, leading to bone erosion in regions where the synovium, bone, and cartilage converge (35). Furthermore, innate immune cells play a significant role in ACPA-negative rheumatoid arthritis, since stromal cells and macrophages collaborate to promote osteoclast development via the RANKL/RANK pathway (36). This connection facilitates bone erosion at the junction of the synovium and periosteal membrane, leading to joint destruction that differs from the antibody-mediated mechanisms observed in ACPA-positive rheumatoid arthritis (37). Cytokine and chemokine signaling pathways are essential for sustaining the inflammatory milieu in the synovium. Depending on the cytokine environment, activated T cells divide into different subsets, such as Th1, Th17, and regulatory T cells (Treg) (38). The immune cells, in conjunction with FLS, perpetuate chronic synovial inflammation and exacerbate the damaging characteristics of ACPA-negative RA (**Figure 2**).

## An overview of NF-κB signaling pathway in RA

**Figure 3:**
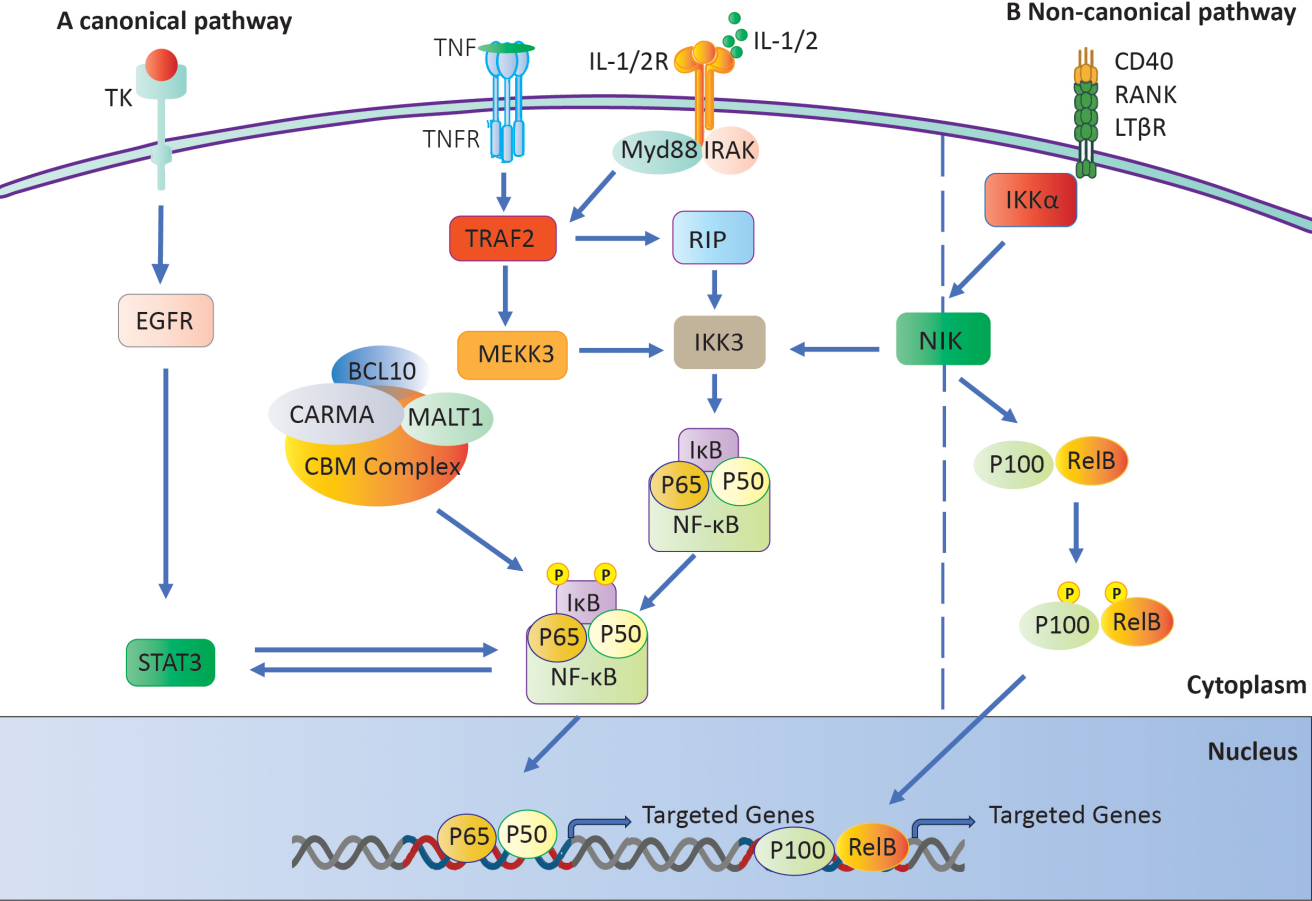
The NF-κB signaling pathway. **(A)** The IKK complex activates the canonical pathway by phosphorylating and degrading IκB, which results in the activation of NF-κB. **(B)** Activation of a Not canonical Pathway: The NF-κB-inducing kinase (NIK) initiates the activation of IKKα, which in turn generates and transports p52 and phosphorylates p100.

Researchers have linked NF-κB to the development of numerous inflammatory disorders, including RA (39, 40). Two distinct pathways can trigger this mechanism: the classical or canonical pathway and the alternative or non-canonical pathway (41). NF-κB subunits first bind to p100 or p105 precursors in the noncanonical pathway. The process then converts these precursors into p50 and p52 subunits, respectively (42). The C-terminal regions of these precursors contain IB-like domains, which impede nuclear localization until they undergo cleavage. The IKK complex activates NF-κB-inducing kinase (NIK), regulating its processing (43). The NF-κB pathway is stimulated in a different way by signals from TNFR superfamily members like BAFFR, CD40, and lymphotoxin receptors (44, 45). There is a key regulatory mechanism that frees NF-κB dimers from IκB. It is made up of IKKα, IKKβ, and the scaffold protein IKKγ/Nemo (46, 47). Subsequently, the liberated IκB molecules undergo degradation by proteasomes (48). The NF-κB dimers move into the nucleus and attach to κB sites on certain genes (43). This alters the expression of those genes and initiates the transcription of genes implicated in immune and inflammatory responses (**Figure 3A**) (48).

The noncanonical pathway primarily involves the IκB family members p100 and p105. When receptors like CD40 and lymphotoxin receptor (LTR) are activated, NIK phosphorylates p100 (49). This causes p100 to go through ubiquitination, which creates p52. Once paired, p52 and RelB move to the nucleus, attaching to B sites to control the expression of genes they are interested in (**Figure 3B**) (50).

NF-κB initiates the production of pro-inflammatory cytokines, including IL-1, TNF-α, and IL-6, in monocytes and macrophages (51). Cytokines have the ability to stimulate NF-κB in both the innate immune system and fibroblast cells. This activation leads to the production of additional cytokines and chemokines, which in turn promote inflammation (51). These compounds then attract other inflammatory cells, contributing to inflammation’s propagation. In RA, the two different canonical and noncanonical NF-κB pathways promote the conversion of monocytes and macrophages into osteoclasts, resulting in bone resorption and inflammatory bone loss (52). NF-κB indirectly enhances Th17 cell formation by inducing IL-1, IL-6, and IL-23 secretion in cells involved in innate immunity. It directly regulates Th17 lineage transcription factor activity in T cells (53, 54). NF-κB activation that isn’t working right also makes it easier for B cells to attack the body’s own tissues and for antibodies to be made that attack the body itself. Both of these things play a part in the development of RA (55, 56). Studies show that people with RA who have high levels of a B-cell activating factor from the TNF family have abnormal, noncanonical NF-κB activation (43, 45, 57, 58). Therefore, NF-κB has an important function in the development of rheumatoid arthritis by influencing multiple cell types (57).

## Non-coding RNAs in rheumatoid arthritis: modulating NF-κB signaling

The expression patterns of ncRNAs vary greatly between immune-mediated diseases and across different cell types and organs. Furthermore, specific ncRNAs frequently exhibit abnormal regulation in various inflammatory and autoimmune disorders (59). Research has consistently shown that certain types of non-coding RNAs, like miRNAs, lncRNAs, and circRNAs, are dysregulated in people with RA (60–62). Additionally, a growing body of evidence has revealed a unique profile of non-coding RNAs that exhibit differential expression in RA, underscoring their potential as dependable biomarkers for diagnosis and therapeutic approaches (59). However, we still don’t fully understand the precise roles and molecular mechanisms of these ncRNAs in the development of RA.

In this summary, we focus on the roles of ncRNAs in RA, with a particular emphasis on miRNAs, lncRNAs, and circRNAs, as documented in the available research. It is very important to understand how these dysregulated ncRNAs affect inflammation and autoimmunity in order to figure out how RA starts. It is crucial to discover the specific genes that are affected by these abnormally produced non-coding RNAs in RA in order to develop reliable indicators and efficient therapies.

## MicroRNAs in rheumatoid arthritis: influencing the NF-κB pathway

MicroRNAs are short RNA molecules, usually 18 to 25 nucleotides long, that regulate gene expression after transcription. miRNAs either accelerate the breakdown of mRNA or inhibit its translation into proteins to achieve this regulation (63). Multiple studies have emphasized the crucial significance of miRNAs in several autoimmune disorders, such as rheumatoid arthritis, systemic lupus erythematosus (SLE), and Sjögren’s syndrome. Nevertheless, the levels of expression and function of miRNAs may vary among distinct disorders (63, 64). miRNAs have a crucial role in a wide range of important biological functions and disease processes. These include controlling the cell cycle, sustaining stem cell populations, facilitating organ development, stimulating angiogenesis, and impacting carcinogenesis (65).

Dysregulation of inflammatory pathways is a key driver of RA, which is defined by chronic inflammation and joint deterioration. In particular, NF-κB signaling stimulation is a key mechanism by which many miRNAs promote these inflammatory processes (promoting inflammation) (**Table 1**) (66). MiR-203 significantly enhances synovial inflammation by boosting the release of pro-inflammatory cytokines such as IL-6 and MMP-1 (67). miR-128-3p enhances NF-κB signaling, which in turn causes heightened inflammatory responses, by downregulating TNFAIP3, a crucial inhibitor of NF-κB (68). Likewise, miR-19b amplifies inflammation in the synovium by increasing NF-κB activity through blocking its negative regulators (69). miR-221-3p increases the production of IL-6 and IL-8 by telling macrophages to change into the pro-inflammatory M1 type (70). In addition, miR-138 enhances the release of pro-inflammatory cytokines via stimulating NF-κB (71). Importantly, miR-16 plays a role in promoting methotrexate resistance in RA by enhancing cell survival and reducing apoptosis, leading to decreased drug sensitivity. This miRNA contributes to the persistence of inflammation and limits the effectiveness of methotrexate, thus promoting disease progression in RA (72). Research indicates that mesenchymal stem cells (MSCs) can mitigate rheumatoid arthritis by lowering miR-548e levels and suppressing IκB translation, which in turn diminishes NF-κB signaling activity and alleviates inflammation (73).

**Table 1:** MicroRNAs implicated in the pathogenesis of RA and their association with the NF-κB signaling pathway.

Main Classification	miRNA	Target Genes	Effect	References
**Promoting Inflammation**	miR-203	IL-6, MMP-1	Enhances synovial inflammation by promoting pro-inflammatory cytokine release	(67)
	miR-128-3p	TNFAIP3	Activates NF-κB, leading to heightened inflammatory responses	(68)
	miR-19b	–	Amplifies inflammation in the synovium by increasing NF-κB activity	(69)
	miR-221-3p	IL-6, IL-8	Induces macrophage polarization to pro-inflammatory M1 type	(70)
	miR-138	Pro-inflammatory cytokines	Enhances the release of pro-inflammatory cytokines via stimulating NF-κB	(71)
	miR-16	Methotrexate resistance pathways	Promotes cell survival and reduces apoptosis, leading to decreased drug sensitivity	(72)
	miR-548e	IκB	Lowered by MSCs, suppresses IκB translation, diminishing NF-κB signaling and alleviating inflammation	(73)
	miR-212/132 miR-99b/let-7e/125a	TNFAIP3, IL15, IGF1R	Promotes bone resorption by enhancing osteoclast formation	(74)
	miR-145-5p	RANKL, MMPs	Enhances osteoclast activity, worsening inflammation and cartilage destruction	(75, 76)
	miR-17-92 and miR-18a	Matrix-degrading enzymes and Pro-inflammatory cytokines	Accelerates joint breakdown and inflammation	(77)
	miR-34a	Th17 cells	Encourages Th17 differentiation, contributing to bone degradation	(78)
	miR-143 and miR-145	IGFBP5, SEMA3A	Enhances FLS survival and motility, leading to increased IL-6 production	(79)
	miR-515-5p	TLR4/JNK pathway, WISP1	Modulates FLS by enhancing cell proliferation and reducing apoptosis	(80)
**Anti-inflammatory Roles**	miR-23a	TNF-α pathway	Reduces production of pro-inflammatory cytokines and downregulates NF-κB activity	(81, 82)
	miR-23b	IKK-α, TAB2, TAB3	Inhibits NF-κB activation and suppresses synthesis of inflammatory mediators	(83)
	miR-22	IL6R	Suppresses rheumatoid arthritis by potentially inhibiting the NF-κB pathway	(84)
	miR-27b	IL-1β	Inhibits cell growth and induces apoptosis by blocking NF-κB signaling	(85)
	miR-17	TNF-α, IL-6, IL-8, MMP-1, MMP-13	Inhibits inflammation by decreasing TNF-α signaling and NF-κB activation	(86)
	miR-496	MMP10	Slows the growth of FLS and accelerates apoptosis by targeting the NF-κB pathway	(87)
	miR-124a	PIK3/NF-κB pathway	Inhibits FLS proliferation and inflammation, promoting apoptosis	(88)
	miR-10a	NF-κB activation	Inhibits inflammation by suppressing NF-κB activation	(89)
	miR-205-5p	MDM2	Suppresses NF-κB, reducing inflammation and promoting tissue repair	(90)
	miR-410-3p	TNF-α, IL-1β, IL-6, MMP-9	Reduces inflammation by inhibiting NF-κB pathway	(91)
	miR-23	TNF-α, IL-1β, IL-8	Decreases inflammation by targeting CXCL12 mRNA and inhibiting NF-κB signaling pathway.	(92)
	miR-31	TLR4	Regulates inflammation by targeting TLR4 and reducing pro-inflammatory markers	(93)
	miR-7-5p	p65	Inhibits TNF-α/NF-κB signaling, reducing inflammatory cytokine production and arthritis severity	(94)
	miR-142-3p	MCP-1, TNF-α, IL-6	Enhances cell survival and reduces pro-inflammatory cytokine synthesis via NF-κB and JNK pathways	(95)
	miR-548a-3p	TLR4/NF-κB pathway	Modulates inflammation in RA and shows negative correlation with inflammatory markers	(96)
	miR-766-3p	NF-κB signaling	Stops NF-κB signaling, lowering inflammatory responses	(97)
	miR-20a	RANKL, TLR4/p38 pathway	Reduces osteoclastogenesis and inhibits bone degradation	(98)
	miR-9	NF-κB1-RANKL pathway	Decreases arthritic damage by suppressing cell proliferation and inflammation	(99)
	miR-146a-5p	–	Reduces osteoclast development and bone degradation	(100–102)
	miR-125a-3p	MAST3, Wnt/β-catenin pathway	Reduces inflammation and limits FLS proliferation	(103)
	miR-27a	FSTL1, TLR4/NF-κB pathway	Inhibits migration and infiltration of FLS, resulting in decreased matrix metalloproteinases and inflammation	(104)

In RA, osteoclastogenesis is a crucial mechanism facilitating bone resorption and joint deterioration. Multiple miRNAs facilitate this process by enhancing the formation and function of osteoclasts, the cells responsible for bone resorption. These miRNAs frequently function by activating inflammatory pathways such as NF-κB or by enhancing the production of molecules that facilitate bone disintegration.

The microRNAs miR-212/132 and miR-99b/let-7e/125a promote bone resorption by increasing osteoclast development and targeting important regulators such as TNFAIP3, IL15, and IGF1R, which are crucial for regulating inflammation and metabolism in bones (74). This is analogous to the function of miR-145-5p, which enhances osteoclast activity and bone degradation. miR-145-5p increases RANKL and MMP levels through NF-κB activation. Its upregulation worsens inflammation and cartilage destruction, making it a potential target for RA treatment (75, 76). By raising the levels of matrix-degrading enzymes and pro-inflammatory cytokines, the miR-17-92 cluster, and miR-18a in particular, makes joints break down faster and cause more inflammation (77). Moreover, miR-34a encourages Th17 cell differentiation, which indirectly causes bone degradation and osteoclast development (78).

In addition, elevated inflammation in RA results in hyperactivation of FLS, which are the main contributors to synovial inflammation and joint deterioration. These cells undergo excessive proliferation due to persistent inflammation, leading to synovial hyperplasia and joint deterioration.

Numerous miRNAs modulate the proliferation, survival, and apoptosis of FLS, thereby affecting the degree of inflammation. Key microRNAs such as miR-143 and miR-145 are upregulated in rheumatoid arthritis fibroblast-like synoviocytes, targeting IGFBP5 and SEMA3A, resulting in elevated IL-6 production, NF-κB activation, and improved fibroblast-like synoviocyte survival and motility (79). miR-515-5p modulates fibroblast-like synoviocytes in rheumatoid arthritis by enhancing cell proliferation and diminishing apoptosis via the TLR4/JNK pathway and the WISP1 gene. The inhibition of miR-515-5p mitigates these effects, indicating its involvement in regulating inflammation and fibroblast-like synoviocyte activity in rheumatoid arthritis (80).

While certain microRNAs have negative impacts on RA, others act as protective agents by lowering cytokine production and downregulating inflammatory pathways, especially NF-κB (anti-inflammatory roles) (**Table 1**). MiR-23a exhibits anti-inflammatory properties in RA by inhibiting TNF-α signaling and reducing the production of pro-inflammatory cytokines, such as IL-6 and IL-8. By targeting the NF-κB pathway (81, 82). Similarly, miR-23b inhibits NF-κB activation and suppresses the synthesis of inflammatory mediators via inhibiting IKK-α, TAB2, and TAB3 (83). Moreover, miR-22 suppresses rheumatoid arthritis by targeting IL6R and potentially inhibiting the NF-κB pathway (84). Furthermore, overexpression of miR-27b stopped cells from growing, sped up cell death, and blocked the NF-κB signaling pathway by focusing on IL-1β (85).

In addition, miR-17 stops inflammation by decreasing TNF-α signaling. This lowers the activation of NF-κB, STAT3, and c-Jun, which in turn lowers the production of pro-inflammatory cytokines like IL-6, IL-8, MMP-1, and MMP-13 (86). Importantly, miR-496 slowed the growth of rheumatoid arthritis fibroblast-like synoviocytes and sped up apoptosis by targeting MMP10 and changing the NF-κB pathway (87). MiR-124a inhibits FLS proliferation and inflammation in RA by targeting the PIK3/NF-κB pathway, reducing cytokine levels, and promoting FLS apoptosis, making it a potential therapeutic target (88). Similarly, miR-10a regulates inflammation by inhibiting NF-κB activation; its suppression leads to increased FLS proliferation and cytokine production, suggesting its role as a target for RA management (89).

Exosome-delivered miR-205-5p targets MDM2 and suppresses NF-κB, which in turn reduces inflammation in the joints and promotes tissue repair (90). Likewise, miR-410-3p reduces inflammation in RA by inhibiting the NF-κB pathway, leading to decreased levels of pro-inflammatory cytokines like TNF-α, IL-1β, IL-6, and MMP-9 (91). Overexpression of miR-23 also decreased inflammation by lowering levels of TNF-α, IL-1β, and IL-8. This was done by targeting CXCL12 mRNA and stopping the NF-κB signaling pathway (92). Interestingly, miR-31 regulates inflammation in RA by targeting TLR4 and reducing pro-inflammatory markers like TNF-α and IL-1, while promoting apoptosis in synovial cells, positioning it as a potential therapeutic target for RA (93). Moreover, miR-7-5p inhibits the TNF-α/NF-κB signaling pathway by binding to the 3’-UTR of p65, resulting in diminished production of inflammatory cytokines and alleviating arthritis severity (94).

miR-142-3p enhances cell survival and diminishes the synthesis of pro-inflammatory cytokines (MCP-1, TNF-α, IL-6) via the NF-κB and JNK pathways. Inhibition of miR-142-3p negates these protective effects (95). In addition, miR-548a-3p is markedly downregulated in rheumatoid arthritis patients and modulates inflammation through the TLR4/NF-κB signaling pathway (96). It exhibits a negative correlation with inflammation markers (CRP, RF, ESR) and contributes to the attenuation of cellular activity and immunological responses. miR-766-3p stops NF-κB signaling, which lowers inflammatory responses in RA (97).

Specific miRNAs reduce inflammation and bone degradation associated with RA by blocking critical inflammatory pathways, therefore safeguarding joint tissues and averting bone loss. These miRNAs function by inhibiting osteoclastogenesis, diminishing the synthesis of pro-inflammatory cytokines, and facilitating the resolution of inflammation. miR-20a reduces osteoclastogenesis and inhibits bone degradation by modulating RANKL via the TLR4/p38 signaling pathway. The injection of agomiR-20a suppresses osteoclast growth and bone erosion, underscoring its potential as a therapeutic target for rheumatoid arthritis to avert bone loss (98). Lee et al. discovered that miR-9 decreases arthritic damage by suppressing the NF-κB1-RANKL pathway, hence decreasing cell proliferation and inflammation (99). Bogunia-Kubik et al. found that the NFkB1 ins/ins genotype is linked to a lower response to TNF-α inhibitor therapy in people with rheumatoid arthritis, as shown by lower levels of miR-146a-5p before treatment. Following therapy, the protective rs2910164-C allele was correlated with elevated miR-146a-5p levels. Furthermore, miR-146a administration to Ly6Chigh monocytes reduced osteoclast development and bone degradation, indicating its potential as a therapeutic target in arthritis (100–102).

Certain microRNAs in RA are essential for modulating inflammation and proliferation of fibroblast-like synovial cells, which are significant contributors to joint destruction in RA. miR-125a-3p reduces inflammation and restricts the proliferation of fibroblast-like synovial cells in rheumatoid arthritis by targeting MAST3 and regulating the Wnt/β-catenin and NF-κB signaling pathways (103). miR-27a, which is downregulated in individuals with rheumatoid arthritis, inhibits the migration and infiltration of FLS via targeting follistatin-like protein 1 (FSTL1). This action results in diminished amounts of matrix metalloproteinases and Rho family proteins, while also inhibiting the TLR4/NF-κB pathway (104).

## Long non-coding RNAs in rheumatoid arthritis: key players in NF-κB signaling

LncRNAs are a recently discovered group of non-coding RNAs that are widely expressed in different human organs and typically consist of more than 200 nucleotides (105). LncRNAs can be categorized into five distinct categories according to their structure and function: sense, antisense, bidirectional, intronic, and intergenic (106). Within the field of oncology, specific lncRNAs exhibit oncogenic characteristics, while others impede the growth and advancement of tumors. These lncRNAs display varied expression patterns and exert distinct biological effects within tumor cells (107). Recent studies have shown that different lncRNAs are expressed at different levels in immune cells in diverse autoimmune illnesses, such as rheumatoid arthritis (63, 108, 109). These results show that distinct lncRNA expression profiles, which may also manifest uniquely in different cells and tissues, distinguish various autoimmune diseases.

Peripheral blood mononuclear cells (PBMCs) of RA patients upregulated the long non-coding RNA (lncRNA) HIX003209, which was associated with TLR2 and TLR4. HIX003209 promotes inflammation by sponging miR-6089, thus enhancing TLR4/NF-κB signaling in macrophages. This lncRNA increased macrophage proliferation and activation, contributing to RA pathogenesis. Therefore, targeting lncRNA HIX003209 could be a potential therapeutic strategy for reducing inflammation in RA (110). Furthermore, RA patients significantly increased LINC00305 expression, which correlates with disease activity markers such as DAS28, C-reactive protein, erythrocyte sedimentation rate, rheumatoid factor, and anti-cyclic citrullinated peptide antibody. The rs2850711 polymorphism in LINC00305 was associated with higher RA susceptibility, especially in individuals with AT and TT genotypes. High levels of LINC00305, NF-κB, and MMP-3 were found in these genotypes. This suggested that LINC00305 and its genetic variant played important roles in diagnosing RA, managing it, and making it worse (111).

Yang et al. discovered that the long non-coding RNA (lncRNA) H19 makes the inflammatory damage caused by TNF-α worse in MH7A cells, which are used to study RA. The levels of H19 went up because of TNF-α. This made TAK1 phosphorylated, which turned on the NF-κB and JNK/p38 MAPK pathways. Silencing H19 reduced inflammatory cytokines (IL-8, IL-1β, and IL-6), while H19 overexpression reversed this effect, indicating that H19 enhances inflammation through the TAK1 pathway in RA (112). Moreover, overexpression of LINC01197 in a mouse RA model reduced disease severity, inhibited fibroblast-like synoviocyte (RA-FLS) proliferation and inflammation, and promoted apoptosis. By sponging miR-150, LINC01197 functioned by increasing THBS2 expression and inactivating the TLR4/NF-κB signaling pathway, thereby reducing inflammation and suggesting LINC01197 as a potential therapeutic target for RA (113). Overexpressing OIP5-AS1 also decreased the severity of RA symptoms and levels of inflammatory factors (IL-1β, IL-10, IL-6, and TNF-α) in a rat model. OIP5-AS1 connects to miR-448 and raises the expression of paraoxonase 1 (PON1). This stops the growth and inflammation of fibroblast-like synoviocytes (RA-FLS). Activation of TLR3 promoted RA progression, while OIP5-AS1 inactivated the TLR3-NF-κB signaling pathway. Therefore, OIP5-AS1 mitigates RA progression through the miR-448-PON1 axis and the TLR3-NF-κB pathway, offering potential for molecularly based RA treatments (114).

Tang et al. found that RA-affected synovial tissues increase the expression of long non-coding RNA (lncRNA) PVT1, while miR-145-5p expression decreases. PVT1 interacts with miR-145-5p and exerts a negative regulatory effect. More PVT1 and less miR-145-5p were found in fibroblast-like synoviocytes from people with rheumatoid arthritis (RA-FLSs) when TNF-α was present. It was possible to silence PVT1 by targeting the NF-κB pathway through miR-145-5p. This decreased TNF-α-induced cell proliferation, increased apoptosis, and decreased the production of inflammatory cytokines (IL-1β and IL-6). These findings indicate that PVT1 has a significant impact on rheumatoid arthritis by regulating cell growth and inflammation via miR-145-5p (115).

Furthermore, the delivery of LncRNA-GAS5 siRNA caused a decrease in joint swelling and a reduction in the quantity of arthritis-related biochemicals, oxidative stress markers, and cytokines in the serum of RA patients. Some other things that were lowered were miR-103, MMP-13, Akt, NF-κB, FGF21, PI3K, and p38 in cartilage. Histopathological analysis showed that LncRNA-GAS5 siRNA ameliorated cartilage pathological changes, suggesting that it prevents cartilage destruction through decreasing miR-103 expression and associated inflammatory pathways (116).

Xiao et al. investigated the function of NEAT1 in RA and discovered its upregulation in RA-FLSs. NEAT1 promotes RA-FLS proliferation and inflammatory cytokine production, while inhibiting apoptosis through miR-204-5p targeting by the NF-κB pathway. Silencing NEAT1 reduced cellular proliferation and inflammation while increasing apoptosis in TNF-α-treated RA-FLSs. These findings suggest that NEAT1 could be a possible therapeutic target for RA by modulating miR-204-5p and the NF-κB pathway (117).

In a different study, it was shown that the exosomal lncRNAHAND2-AS1 stops the activation of RA-FLS. By blocking miR-143-3p and raising TNFAIP3 expression, HAND2-AS1 lowers RA-FLS growth, migration, and inflammation and speeds up cell death through the NF-κB pathway. This suggests that MSC-derived exosomal HAND2-AS1 could be a potential therapeutic approach for RA (118). Furthermore, linc00152 was upregulated in RA-FLS and stimulated by TNF-α/IL-1β through the NF-κB pathway. Linc00152 caused inflammation by blocking miR-103a, which increased TAK1 expression and turned on the NF-κB pathway that is controlled by TAK1. Additionally, NF-κB enhanced linc00152 expression via the transcription factor YY1. This linc00152/NF-κB feedback loop exacerbated RA-FLS inflammation, suggesting that linc00152 could be a potential diagnostic and therapeutic target for RA (119).

Liu et al. investigated the role of lncRNA XIST in RA and found it to be significantly upregulated in RA synovial tissues and cells. By sponging miR-126-3p, XIST promotes cell proliferation and inhibits apoptosis in RA-FLS. Lowering XIST levels raised miR-126-3p levels, which blocked the NF-κB pathway by lowering p-p65 and p-IκBα expression. This caused RA-FLS cells to multiply less and die more. This study suggests that targeting the XIST/miR-126-3p/NF-κB axis could be a potential therapeutic approach for RA (120).

Another study showed that linc00324 increased the number of CD4+ T cells and the release of MIP-1α by turning on the NF-κB pathway. This was achieved by targeting and sponging miR-10a-5p, which normally inhibits CD4+ T cell proliferation and NF-κB activation. It’s possible that linc00324 makes RA inflammation worse through the miR-10a-5p/NF-κB axis because overexpressing miR-10a-5p reversed its pro-inflammatory effects. This identified LINC00324 as a potential therapeutic target for RA (121). However, RA-FLSs and peripheral blood mononuclear cells (PBMCs) from RA patients downregulate the lncRNA MAPKAPK5-AS1 (MK5-AS1). By connecting to miR-146a-3p and controlling SIRT1 expression, MK5-AS1 overexpression in RA-FLSs reduced inflammation and boosted apoptosis. This was done by affecting the NF-κB pathway. These effects were reversed by SIRT1 knockdown or miR-146a-3p overexpression. The study also revealed that WTAP downregulation promoted MK5-AS1 RNA stability. This research suggests that MK5-AS1, via the miR-146a-3p/SIRT1/NF-κB axis, could be a potential therapeutic target for RA (122).

Sun et al. discovered that the long non-coding RNA AL928768.3 encourages the growth, invasion, and inflammation of RA-FLS and stops cell death by turning on the LTB-mediated NF-κB pathway. Overexpression of AL928768.3 increased IL-1β, IL-6, IL-8, and LTB levels, as well as NF-κB signaling proteins, while knockdown had the opposite effect, which could be reversed by LTB overexpression. This highlights AL928768.3 as a potential therapeutic target for RA (**Table 2**) (123).

**Table 2:** A brief summary of the main discoveries associated with long noncoding RNAs in RA.

lncRNA	Role in RA	Regulation Status	Target/Pathway	Ref.
HIX003209	Promotes inflammation by sponging miR-6089, enhancing TLR4/NF-κB signaling in macrophages	Upregulated in PBMCs of RA patients	TLR4/NF-κB pathway	(110)
LINC00305	Correlates with disease activity markers and RA susceptibility	Upregulated in RA patients	NF-κB pathway	(111)
H19	Exacerbates TNF-α-induced inflammatory injury, promotes TAK1 phosphorylation, activating NF-κB and JNK/p38 MAPK pathways	Upregulated in TNF-α treated cells	TAK1/NF-κB and JNK/p38 MAPK pathways	(112)
LINC01197	Reduces disease severity, inhibits RA-FLS proliferation and inflammation, promotes apoptosis	Overexpressed in a mouse RA model	miR-150/THBS2/TLR4/NF-κB pathway	(113)
OIP5-AS1	Reduces symptom severity and inflammatory factor levels, inhibits RA-FLS growth and inflammation	Overexpressed in a rat RA model	miR-448/PON1/TLR3-NF-κB pathway	(114)
PVT1	Upregulated in RA synovial tissues, binds and negatively regulates miR-145-5p	Upregulated in RA synovial tissues	miR-145-5p/NF-κB pathway	(115)
GAS5	Reduces joint swelling, serum levels of arthritis-related biochemicals, cytokines, and oxidative stress markers	Reduced in RA cartilage	miR-103/NF-κB pathway	(116)
NEAT1	Promotes RA-FLS proliferation and inflammatory cytokine production, inhibits apoptosis	Upregulated in RA synovial tissues and cells	miR-204-5p/NF-κB pathway	(117)
HAND2-AS1	Inhibits RA-FLS activation by sponging miR-143-3p and increasing TNFAIP3 expression	Upregulated in MSC-derived exosomes	miR-143-3p/NF-κB pathway	(118)
linc00152	Promotes inflammation by inhibiting miR-103a, leading to increased TAK1 expression and activation of the TAK1-mediated NF-κB pathway	Upregulated in RA-FLS	miR-103a/TAK1/NF-κB pathway	(119)
XIST	Promotes cell proliferation and inhibits apoptosis in RA-FLS by sponging miR-126-3p	Upregulated in RA synovial tissues and cells	miR-126-3p/NF-κB pathway	(120)
linc00324	Promotes CD4+ T cell proliferation and MIP-1α secretion by targeting and sponging miR-10a-5p	Upregulated in RA CD4+ T cells	miR-10a-5p/NF-κB pathway	(121)
MK5-AS1	Inhibits inflammation and promotes apoptosis in RA-FLS by binding to miR-146a-3p and regulating SIRT1 expression	Downregulated in RA-FLS and PBMCs	miR-146a-3p/SIRT1/NF-κB pathway	(122)
AL928768.3	Promotes proliferation, invasion, and inflammation in RA-FLS, inhibits apoptosis	Upregulated in RA-FLS	LTB/NF-κB pathway	(123)

## Circular RNAs in rheumatoid arthritis: controlling NF-κB signaling

**Figure 4:**
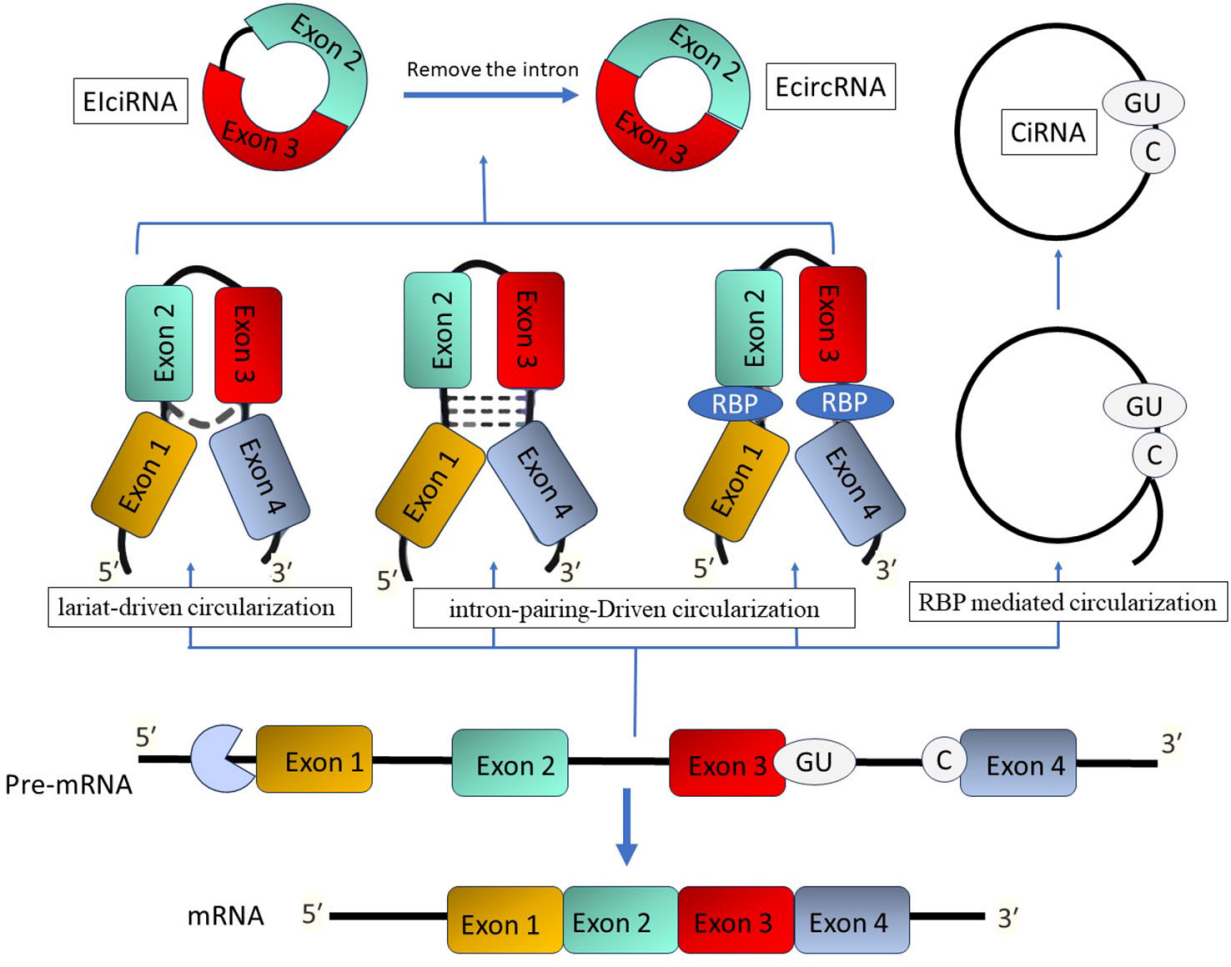
The process of circular RNA formation. There are three methods that give rise to circular RNAs: lariat-driven, intron-pairing-driven, and RBP-mediated circularization. Endogenous inverted repeat-derived circular RNAs (EIciRNAs) are composed of conserved exons and introns. Splicing generates circular intronic RNAs (ciRNAs) that possess a 7-nucleotide GU-rich region adjacent to the 5′ splice site and an 11-nucleotide C-rich motif at the branch point.

The unique covalently closed loop structure of circRNA makes it stand out from other non-coding RNAs. It doesn’t have any 5′ end caps or 3′ polyadenylate tails (124). Circular RNA molecules often possess a circular structure, which provides them with stability and often results in a half-time of more than 48 hours (125). CircRNAs are categorized into three primary types: exonic circRNAs (ecircRNAs), circular intronic RNAs (ciRNAs), and exon-intron circRNAs (EIciRNAs) (126). Their synthesis in cells usually occurs due to exon skipping and subsequent circularization, which can be assisted by intron pairing or the activity of RNA-binding proteins (126). C CircRNAs have been detected not just in mammals but also in fungi, plants, and protists. These molecules are predominantly expressed in a tissue-specific way and can be identified in peripheral blood, exosomes, and different organs (**Figure 4**) (127, 128).

CircRNAs have the ability to behave as miRNA decoys, sequestering miRNAs and inhibiting their interaction with their endogenous mRNA targets, similar to lncRNAs (129). The interaction mainly takes place in the cytoplasm, where ecircRNAs might have important functions in several pathological and physiological processes through the competitive endogenous RNA (ceRNA) mechanism (130). In contrast, ciRNAs and EIciRNAs primarily influence gene expression within the nucleus. CircRNAs have a role in regulating the expression of target messenger RNAs (mRNAs) by absorbing miRNAs. This contact, known as ceRNA interaction, has an impact on several processes such as autoimmune and inflammation (126, 129). Nevertheless, the intricate mechanisms underlying the ceRNA actions of circRNA in RA have not been well investigated.

Circular RNAs play a role in the control of several immune-related illnesses by acting as miRNA sponges or reservoirs (126). Studies have emphasized the substantial impact of circRNAs in a wide range of diseases, such as cancer, disorders of the nervous system, and cardiovascular diseases (131, 132). Their crucial role in antiviral immunity has been firmly established, offering possible therapeutic possibilities by focusing on circRNAs for immunologic treatments (133, 134).

Yang et al. discovered a notable increase in the expression of circRNA_09505 in the peripheral blood mononuclear cells of individuals with RA. This circular RNA enhances macrophage growth and secretion of cytokines (TNF-α, IL-6, and IL-12) by acting as a miR-6089 inhibitor, resulting in the stimulation of the AKT1/NF-κB signaling pathway. In mice with collagen-induced arthritis (CIA), CircRNA_09505 knockdown reduced arthritic symptoms and inflammation, indicating its contribution to the worsening of rheumatoid arthritis (135).

Furthermore, overexpressing circ-Sirt1 inhibited proliferation and induced apoptosis in RA-FLS. This circRNA lowered the amounts of inflammatory cytokines (IL-6, IL-1β, and TNF-α) and matrix metalloproteinases (MMP1 and 3) while raising the levels of Sirt1 and Nrf2. This decreased oxidative stress and inflammation. The protective effects were mediated through miR-132, which enhanced the Sirt1 pathway, highlighting Circ-Sirt1’s potential therapeutic role in RA (136).

Furthermore, it has been demonstrated that circ_0088036 is elevated in both the sera and RA-FLS cells of individuals with rheumatoid arthritis. This upregulation of circ_0088036 contributes to the stimulation of cell proliferation, advancement of the cell cycle, and activation of inflammatory responses by acting as a sponge for miR-1263. This interaction resulted in the overexpression of REL, a constituent of the NF-κB pathway, hence augmenting NF-κB signaling. The specific suppression of circ_0088036 hindered the growth of RA-FLS cells and triggered programmed cell death, indicating its involvement in the development of rheumatoid arthritis through the miR-1263/REL/NF-κB pathway (137).

**Figure 5:**
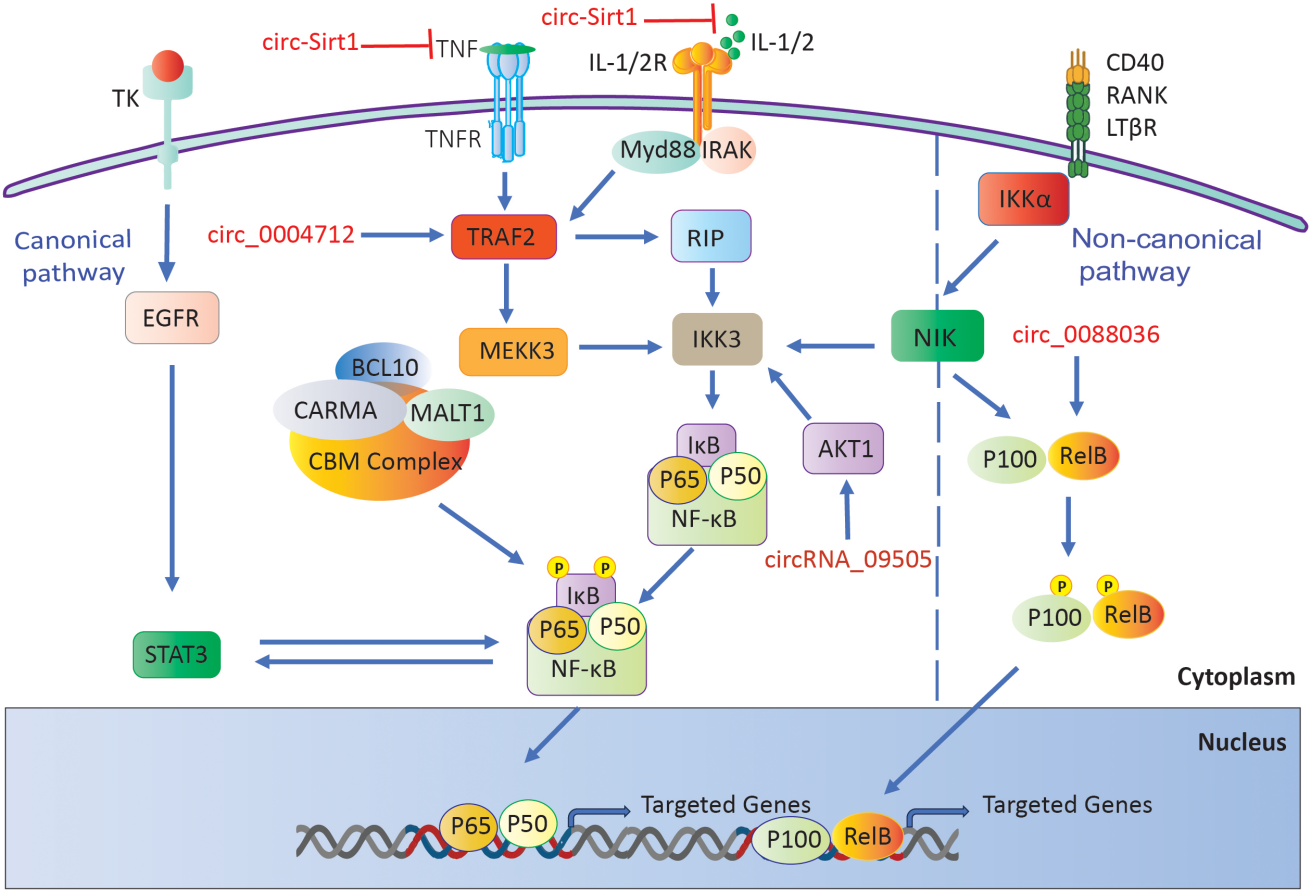
RA-associated circRNAs influence the NF-κB signaling pathway.

A separate investigation revealed that circ_0004712 exhibited increased expression in both RA synovial tissues and RA-FLS. Researchers found that the upregulation of circ_0004712 stimulates cell proliferation, migration, and inflammation while simultaneously suppressing apoptosis. This circular RNA acts as a sponge for miR-633, resulting in an increase in TRAF6 expression, which in turn activates the NF-κB signaling pathway. Knocking down circ_0004712 led to a decrease in aggressive behaviors in RA-FLS, suggesting its involvement in RA progression through the miR-633/TRAF6/NF-κB pathway (**Figure 5**) (138).

## NcRNA-based therapies for RA: new biomarkers and treatment approaches

In recent years, there has been a significant rise in the investigation of the potential of ncRNAs as biomarkers in RA (139). Nevertheless, the development of ncRNA-based therapeutics for RA is still in the beginning stages. miRNA-based therapies, which include antagonists that lower miRNA expression, promote RA, and mimics that restore the activity of miRNAs that suppress RA, are some of the most promising approaches (140). For instance, studies have shown that methotrexate increases the levels of miR-877-3p, leading to a decrease in GM-CSF and CCL3, thereby restricting the growth and migration of synoviocytes (141). Similarly, Resolvin D1 raises CTGF by increasing miR-146a-5p‐85, and berberine, a chemical found in plants, stops FLS growth and bone loss by targeting Wnt/β-catenin and RANKL-mediated pathways (142).

Another strategy is to modify miRNAs’ upstream regulators in the ceRNA network. In this complex structure, circRNAs and lncRNAs function as molecular sponges. By manipulating these substrates, it is possible to alter the activity of miRNAs and the results of RA (140). As an example, astragaloside lowers the lncRNA LOC100912373, which then activates miR-17-5p’s blocking of PDK1 and limits the growth of FLS (143). Paeoniflorin regulates the circ-FAM120A/miR-671-5p axis, inhibiting inflammation, cell cycle progression, and synoviocyte proliferation (144).

The ceRNA network is also a mechanism through which certain miRNA antagonists’ function. As an example, the triptolide-controlled hsa-circ-0003353/miR-31-5p/CDK1 axis and tocilizumab’s impact on lncRNA MIR31HG/miR-214/PTEN/AKT improve miRNA sponges, which leads to less inflammation, cell cycle arrest, and cellular proliferation (145, 146). Recognized mechanisms enhance the respective miRNA sponges and alleviate the suppression of miRNAs on their target genes. This leads to diminished cell cycle arrest, cellular proliferation, and reduced production of inflammatory mediators.

Furthermore, using plant-derived miRNAs as dietary interventions is a promising approach to RA therapy (147). Despite the potential for safety and convenience, this method faces obstacles in terms of the optimal plant miRNA types, their stability in the body, and the interval of treatment (147).

Advances in miRNA delivery through tissue engineering technologies have made new therapeutic options possible. Exosomes are particularly promising due to their biocompatibility and targeted delivery capabilities, while synthetic nanoparticles and cationic liposomes are extensively utilized to improve miRNA uptake and stability (148). Exosomes encapsulate miRNAs, protecting them from degradation in the extracellular environment. A thorough screening of plasma exosomes from RA patients discovered 14 dysregulated miRNAs. Among them, miR-204-5p was significantly downregulated and inversely linked with important clinical markers like ESR, RF, and CRP. FLS proliferation is inhibited by exosomes derived from T cells that contain miR-204-5p, suggesting a potential therapeutic function (148).

Exosomes derived from MSCs also exhibit substantial therapeutic potential. For example, bone marrow MSC exosomes (BM-MSC-EVs) containing miR-34a activate the p53 signaling pathway, thereby inducing apoptosis and suppressing FLS proliferation (149). In the same vein, BM-MSC-EVs deliver miR-223 to the NLRP3 inflammasome, thereby reducing inflammation. The therapeutic strategy is attractive due to the reduced risk of rejection associated with the use of autologous MSC-derived exosomes (150).

Furthermore, miRNA profiling has the potential to revolutionize RA treatment through precision medicine, providing new opportunities for personalized therapies and enhanced patient outcomes. This is particularly true for drug validation and early diagnosis. Continued research into miRNA-based therapeutics will provide innovative, highly targeted treatment options in the future, revolutionizing RA management (151).

Understanding the hub’s function in normal regulation is just as important as identifying it to target critical pathways in the miRNA network for treatment. For instance, both miR-20a and miR-26b can target the NF-κB signaling pathway (152). Nevertheless, NF-κB also serves physiological purposes by facilitating the typical immune response (153). Normal immune response will be compromised if the inflammatory process and immune cells are completely inhibited. Consequently, it is imperative to resolve the critical issue of achieving equilibrium or shifting the balance in favor of RA treatment. Furthermore, studies have shown that the genes targeted by miR-223 have opposing effects on FLS cells. Some of its effects include reducing inflammation and inducing apoptosis in FLS cells by targeting NLRP3 (154), and increasing FLS proliferation and arthritis by targeting FOXO1 (155). It is possible that the non-standardization of research circumstances and baseline variables is responsible for these contradictory results. The miRNA panel may help shed light on the molecular processes of RA in different cell types and make RA diagnoses easier. Through the use of miRNAs in cancer to trigger cell death and prevent blood vessel formation, or FLS hyperplasia, it may also provide new information and ideas for future studies. As our comprehension of the subject expands, conducting a more thorough investigation of the miRNAs in RA patients may provide additional insights into diagnosis and treatment (140). Furthermore, miRNA profiling has the potential to transform RA treatment through precision medicine, thereby enabling the development of personalized therapies and improved patient outcomes. This is especially true in the context of drug validation and early diagnosis. In the future, innovative, highly targeted treatment options will be provided by ongoing research into miRNA-based therapeutics, which will revolutionize RA management (156).

## Conclusions

Non-coding RNAs play a key role in controlling the NF-κB signaling pathway, and this study looked into how they affect the progression of rheumatoid arthritis. Non-coding RNAs, such as microRNAs, long non-coding RNAs, and circular RNAs, profoundly affect inflammatory and immunological responses essential for the therapy of RA. Our work has contributed to the comprehension of the functional dynamics of these ncRNAs, emphasizing their potential as biomarkers and therapeutic targets. NcRNAs’ complex control of the NF-κB pathway facilitates novel therapeutic techniques that may improve RA treatment’s precision and efficacy. The increasing interest in ncRNA-based therapies presents opportunities for novel treatment approaches. However, we must resolve obstacles related to efficient gene regulation and delivery to fully realize their therapeutic advantages.
